# Phenotypic and Proteomic Insights into Differential Cadmium Accumulation in Maize Kernels

**DOI:** 10.3390/genes14122204

**Published:** 2023-12-13

**Authors:** Huanle Guo, Min Deng, Feng Yu, Han Li, Zhongyang Cao, Qiang Zeng, Zhihui Chen, Hongbing Luo, Bin Tang

**Affiliations:** 1 College of Agronomy, Hunan Agricultural University, Changsha 410128, China; guohuanle@hunaas.cn (H.G.); dengmin@hunau.edu.cn (M.D.); 2 Crop Research Institute, Hunan Academy of Agricultural Sciences, Changsha 410125, China; lihan@hunaas.cn (H.L.); caozhongyang@hunaas.cn (Z.C.); zengqiang@hunaas.cn (Q.Z.); czh2008@hunaas.cn (Z.C.); 3 Maize Engineering Technology Research Center of Hunan Province, Changsha 410128, China; 4 State Key Laboratory of Biocatalysis and Enzyme Engineering, School of Life Sciences, Hubei University, Wuhan 430062, China; yufeng@hubu.edu.cn

**Keywords:** maize, cadmium, proteomics, kernel, mutant

## Abstract

The contamination of agricultural soil with cadmium (Cd), a heavy metal, poses a significant environmental challenge, affecting crop growth, development, and human health. Previous studies have established the pivotal role of the *ZmHMA3* gene, a P-type ATPase heavy metal transporter, in determining variable Cd accumulation in maize grains among 513 inbred lines. To decipher the molecular mechanism underlying mutation-induced phenotypic differences mediated by *ZmHMA3*, we conducted a quantitative tandem mass tag (TMT)-based proteomic analysis of immature maize kernels. This analysis aimed to identify differentially expressed proteins (DEPs) in wild-type B73 and *ZmHMA3* null mutant under Cd stress. The findings demonstrated that *ZmHMA3* accumulated higher levels of Cd compared to B73 when exposed to varying Cd concentrations in the soil. In comparison to soil with a low Cd concentration, B73 and *ZmHMA3* exhibited 75 and 142 DEPs, respectively, with 24 common DEPs shared between them. *ZmHMA3* showed a higher induction of upregulated genes related to Cd stress than B73. Amino sugar and nucleotide sugar metabolism was specifically enriched in B73, while phenylpropanoid biosynthesis, nitrogen metabolism, and glyoxylate and dicarboxylate metabolism appeared to play a more significant role in *ZmHMA3*. This study provides proteomics insights into unraveling the molecular mechanism underlying the differences in Cd accumulation in maize kernels.

## 1. Introduction

With the rapid expansion of modern industries, agriculture, and other human activities, a significant volume of chemical products are being released into the environment, leading to severe environmental pollution [1]. Among these pollutants, heavy metals, particularly cadmium (Cd), emerge as a critical threat to crop growth and food safety, with Cd-induced soil pollution being a global issue [2]. Cd enters the environment through various channels such as nickel–Cd batteries, mining, metal smelting, urban pollution, and excessive use of compound fertilizers [3]. While Cd is a non-essential element in crops, it is absorbed by plants alongside other mineral ions, accumulating in plants and causing toxic effects, including physiological and morphological changes [4].

The impact of Cd on plants includes inhibiting seed germination [5], seedling growth [6], and root development [7], disrupting the absorption of other minerals, and inducing plant malformation [8]. Cd also triggers oxidative stress reaction in plants, leading to cell stability destabilization, reduced leaf chlorophyll content, impaired photosynthesis, and ultimately reduced crop yield and quality [4,9]. Under Cd stress, intracellular oxidative stress is intensified, causing protein oxidation, oxidative degeneration of cell membrane phospholipids, and damage to the DNA and RNA, which can ultimately result in cell death [9,10]. In response to these challenges, plants have evolved antioxidant systems and regulatory measures to eliminate reactive oxygen species and maintain normal cell function [11].

Cd accumulation varies among plant species, and various crops have evolved functions to reduce Cd uptake or mitigate its toxic effects. Rice accumulates more Cd than maize, barley, sorghum, and other species [12]. *OsHMA3* functions as a Cd transporter in rice, facilitating the transfer of Cd to the root vacuole. Its roles include restricting Cd transfer from the roots to the stems and leaves, ultimately leading to a reduction in Cd content within the grain [13]. *OsNramp1* [14] and *OsNramp5* [15] serve as primary proteins responsible for Cd absorption in rice roots. The loss of their function significantly diminishes Cd content in the grain. *CAL1* operates by chelating Cd within the cytosol, facilitating Cd secretion to extracellular spaces, thereby lowering cytosolic Cd concentration [16]. Despite progress in understanding Cd accumulation in rice, little is known about the molecular mechanisms in maize, barley, wheat, and other crops in the last decade [12]. In a previous study, we identified the *ZmHMA3* gene, which controls Cd accumulation in maize grain by regulating Cd uptake and transport. Mutation of this gene leads to a rapid increase in Cd content in maize grain [17]. However, the role of this gene in Cd accumulation in different parts of the maize plant remains understudied.

Proteomics has witnessed rapid advancements, becoming a prominent strategy to elucidate the regulation mechanism of biotic and abiotic stress [18,19,20] and complex traits [21]. While proteomic studies on Cd stress responses have been conducted in various crops [22,23,24,25,26,27,28], investigations on the effects of increased Cd content on the grain proteome and regulation of grain proteins in response to Cd stress are lacking. Proteomic studies can offer insights into the expression characteristics of stress-related proteins during maize grain development, providing valuable information for analyzing the molecular regulation mechanism of the maize grain defense system, enhancing resilience, and improving grain yield and quality [29].

Maize, a vital food and feed crop, plays a crucial role in sustaining human life and health due to its widespread cultivation, adaptability, high yield, and resilience [30]. However, it is susceptible to Cd stress, and the safety of maize products is directly linked to food safety [31]. While previous studies have focused on maize’s vegetative organs’ response to Cd stress, such as the roots and leaves, there is a notable gap in understanding of how its kernels respond to Cd stress. Maize kernels, as harvesting organs, directly influence crop yield, quality, and economic benefits [32,33]. Hence, studying the response of maize kernels to Cd stress is of utmost significance for obtaining a comprehensive understanding of Cd’s effects on crops.

By investigating the dynamic changes in grain Cd content among different Cd accumulation varieties 10–60 days after the pollination period, we observed the highest grain Cd content on the 10th day after pollination, decreasing by approximately half on the 20th day after pollination, followed by a gradual decline with kernel development (unpublished data). Consequently, we identified the period of 10–20 days after pollination as the sensitive period for maize grain response to Cd stress. This study aims to unveil a mechanistic understanding of Cd accumulation differences in maize grains and identify crucial gene targets for breeding Cd-tolerant varieties through morphological, physiological, and proteomic approaches.

## 2. Materials and Methods

### 2.1. Plant Materials and Cd Treatment

The B73 EMS mutant, *ZmHMA3*, carrying nonsense mutation sites in the *ZmHMA3* coding region was sourced from the maize EMS mutant library (http://www.elabcaas.cn/memd/, accessed on 10 January 2022). Validation of the mutation sites was performed by sequencing PCR products with specific primers [17]. B73 (wild-type) and *ZmHMA3* (mutant-type) were bred by the Hunan Academy of Agricultural Sciences. Maize kernels for protein extraction and Cd content detection were collected from B73 and *ZmHMA3*, grown in Changsha (28.20 N, 113.09 E) during the spring of 2021 (20 March to 1 August). The soil chemical properties in the top 20 cm of soil with a low Cd concentration were as follows: pH, 4.42; total Cd, 0.31 mg∙kg^−1^; available Cd, 0.18 mg∙kg^−1^; total N, 1.12 g∙kg^−1^; total P, 1.43 g∙kg^−1^; total K, 10.77 g∙kg^−1^; organic matter, 20.62 g∙kg^−1^; alkali hydrolysable N, 98.14 mg∙kg^−1^; Olsen-P, 24.13 mg∙kg^−1^; and exchangeable K, 141.34 mg∙kg^−1^. In high-Cd-concentration soil, the properties were as follows: pH, 4.41; total Cd, 1.83 mg∙kg^−1^; available Cd, 1.26 mg∙kg^−1^; total N, 1.24 g∙kg^−1^; total P, 1.13 g∙kg^−1^; total K, 19.12 g∙kg^−1^; organic matter, 21.31 g∙kg^−1^; alkali hydrolysable N, 97.26 mg∙kg^−1^; Olsen-P, 14.96 mg∙kg^−1^; and exchangeable K, 218.42 mg∙kg^−1^. B73 and *ZmHMA3* were planted in two rows with three replicates, using a randomized complete block design. Each plot consisted of 50 plants in 5 m rows, separated by 60 cm. Field management, including fertilization, irrigation, and pest and weed control, followed conventional farming practices.

### 2.2. Morphological and Physiological Parameters

During the harvesting stage, five plants located in the center of each row were sampled, representing one biological replication. Our study involved three biological replications. The collected plant samples, including the roots, stems, leaves, husks, cobs, and kernels, underwent a preliminary drying step at 105 °C for 30 min and were then weighed after further oven-drying at 80 °C until a constant weight was achieved. On the 15th day after pollination, a total of 18 plants were assessed regarding their photosynthetic rate-related parameters. The LI-6400 portable leaf gas exchange system (LICOR Biosciences, Lincoln, NE, USA) was employed to measure parameters such as the net photosynthetic rate, stomatal conductance, intercellular CO_2_ concentration, and transpiration rate of the flag leaves. The SPAD 502 Plus chlorophyll meter (Konica Minolta, Tokyo, Japan) was used to determine the total chlorophyll content of the flag leaves.

The antioxidant enzyme activity indices in each treatment consisted of three biological replicates and three technical replicates. Following the manufacturer’s instructions provided by the Nanjing Jiancheng Biotechnology Co., Ltd. (Nanjing, China), antioxidant enzyme activities were assessed using a plant malondialdehyde (MDA) assay kit (A003-1, TBA thiobarbituric acid method), a plant total superoxide dismutase (T-SOD) assay kit (A001-1, hydroxylamine method), a plant peroxidase (POD) assay kit (A084-3), and a plant catalase (CAT) assay kit (A007-1-1, visible spectrophotometry method).

### 2.3. Determination of Cd Concentrations and Soil Physiochemical Properties

All plant samples were weighed and ground into a fine powder capable of passing through a 100-mesh sieve using a pulverizing machine. A sample consisting of 0.1–0.5 g of powder was collected and digested with HNO_3_/HClO_4_ (9:1, *v*/*v*). Cd concentrations in the roots, stems, leaves, husks, cobs, and kernels were determined using the ZEEnit700P atomic absorption spectrometer (Analytikjena, Jena, Germany). Soil Cd content was measured using a PinAAcle 900T atomic absorption spectrometer (PerkinElmer, Waltham, MA, USA) in accordance with the National Standards of the People’s Republic of China (GB/T 17141-1997) [34]. Soil available cadmium (ACd) was extracted with calcium chloride [34]. Soil pH was measured using a pH meter (PB-10, Sartorious, Göttingen, Germany). Soil physiochemical properties were determined following the methods described by Li et al. [35], which included soil organic matter and available nitrogen (volumetric method), available phosphorus (UV-Vis spectrophotometry), and available potassium (inductively coupled plasma-atomic emission spectrometry).

### 2.4. Sample Collection and Proteomics Analysis

Before silk emergence, female flowers were uniformly covered with paper bags, and hand pollination was conducted on the day after silk emergence. Fifteen days after pollination, ears from five randomly selected plants were chosen, and the kernels from the middle of each ear were stripped and combined to form one biological replicate. The collected grains were pre-cooled in liquid nitrogen and then stored at −80 °C until further processing. For the proteomic study, three independent biological replicates for each treatment of the two genotypes were utilized, totaling 12 kernel samples.

Following the methodology outlined by Jian et al. [25], the experimental procedure for quantitative proteomic analysis included steps such as protein extraction, trypsin digestion, tandem mass tag (TMT) labeling, high-performance liquid chromatography fractionation, liquid chromatography-tandem mass spectrometry analysis, and secondary mass spectrometry data analysis. This analysis was conducted by PTM-Biolabs Co., Ltd. (Hangzhou, China). The mass spectrometry proteomics data have been deposited to the ProteomeXchange Consortium via the PRIDE partner repository with the dataset identifier PXD047578. The repeatability between samples was assessed using the Pearson correlation coefficient and principal component analysis (PCA). In the protein screening process, a fold change greater than 1.3 (up-regulated) or less than 0.77 (down-regulated) at *p* < 0.05 was considered as the threshold for identifying differentially expressed proteins (DEPs) between samples.

### 2.5. Bioinformatics Analysis

Gene Ontology (GO) annotation of the proteome was conducted using the UniProt-GOA database (http://www.ebi.ac.uk/GOA/, accessed on 10 May 2022) and InterProScan 5 software. The DEPs were categorized into biological processes, cellular compartments, and molecular functions. In each category, a two-tailed Fisher’s exact test was employed to assess the significance of DEPs compared to the background of the identified proteins. For further classification and grouping of the DEPs, the Kyoto Encyclopedia of Genes and Genomes (KEGG) database (http://www.genome.jp/kegg/, accessed on 18 May 2022) was utilized to annotate protein pathways based on the protein sequence alignment method. The results were analyzed using a two-tailed Fisher’s exact test to evaluate the enrichment of DEPs relative to all identified proteins. The enriched DEPs were then mapped onto the KEGG pathway database using the KEGG online service tool KEGG mapper. Significant GO and KEGG enrichment analyses of DEPs with a corrected *p*-value < 0.05 were considered. The accession or sequence of DEPs was searched against the protein–protein interaction database STRING (v.11.0), considering a confidence score greater than 0.7 (high confidence). To visualize the differential protein interactions in the resulting interaction network, the R Package ‘networkD3’ tool was employed.

### 2.6. Statistical Analysis

Data processing utilized Microsoft Excel 2010, and statistical analysis was performed using SAS 9.2 (Sas Institute, Cary, NC, USA, 2009). The statistical differences between pairs of means were determined using Duncan’s multiple range test or a two-sided Student’s *t*-test at *p* < 0.05. The translocation factor (TF) of Cd from organ “a” to organ “b” was calculated as follows [36]: TF a-b = Cd concentration in organ “b” (mg/kg)/Cd concentration in organ “a” (mg/kg). Additionally, GraphPad Prism 8.0.0 (GraphPad Software, San Diego, CA, USA) was employed for figure creation.

## 3. Results

### 3.1. Higher Cd Content in ZmHMA3 Than B73

**Figure 1:**
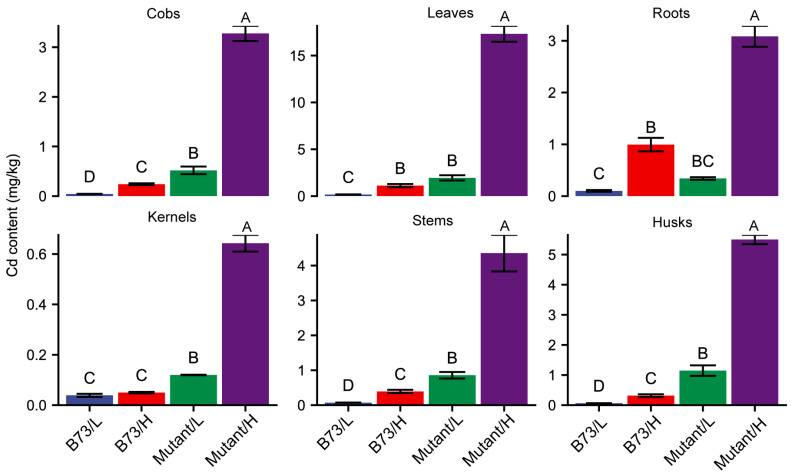
The Cd content in different organs of B73 and the mutant. L represents the low-Cd condition, H represents the high-Cd condition, Mutant represents the genotype *ZmHMA3*, and Cd represents cadmium. The upper letter in each column indicates the difference derived from Duncan’s multiple range test.

To assess the disparity in Cd accumulation between wild-type B73 and mutant-type *ZmHMA3* under varying Cd concentrations in the soil, we measured the Cd content of the roots, stems, leaves, husks, cobs, and kernels (Figure 1). In B73, the Cd content in different organs ranged from 0.039 to 0.164 mg/kg and from 0.050 to 1.114 mg/kg under low- and high-Cd-concentration soil conditions, respectively. Conversely, in *ZmHMA3*, the Cd content in different organs ranged from 0.120 to 1.946 mg/kg and from 0.643 to 17.320 mg/kg under low- and high-Cd-concentration soil conditions, respectively. While the Cd content in the roots of *ZmHMA3* was higher than in those of B73, the difference was not statistically significant. However, the Cd content in the stems, leaves, husks, cobs, and kernels of *ZmHMA3* was significantly higher than in those of B73. High Cd stress significantly influenced the Cd content in different organs of *ZmHMA3*. In B73, no significant differences in kernel Cd content were observed between high- and low-Cd soil conditions, whereas significant differences were noted in the roots, stems, leaves, husks, and cobs under varying soil Cd concentrations.

Regarding Cd translocation factors, both B73 and *ZmHMA3* exhibited a trend of lower Cd translocation in a high Cd concentration compared to a low Cd concentration across various organs (Figure 1). Moreover, in most organs, the Cd translocation factors of *ZmHMA3* were significantly higher than those of B73. This suggests that the high-Cd-accumulating genotype *ZmHMA3* possesses a more robust Cd transport ability from roots to aboveground compared to the low-Cd-accumulating genotype B73. Among all organs subjected to Cd stress, the roots displayed the highest increase in Cd content, while the kernel exhibited the lowest increase. These findings highlight the maize grains as having the most significant difference in Cd accumulation, prompting their selection for further proteomic analysis.

### 3.2. Impact of Soil Cd Concentration on Plant Growth Characteristics

**Figure 2:**
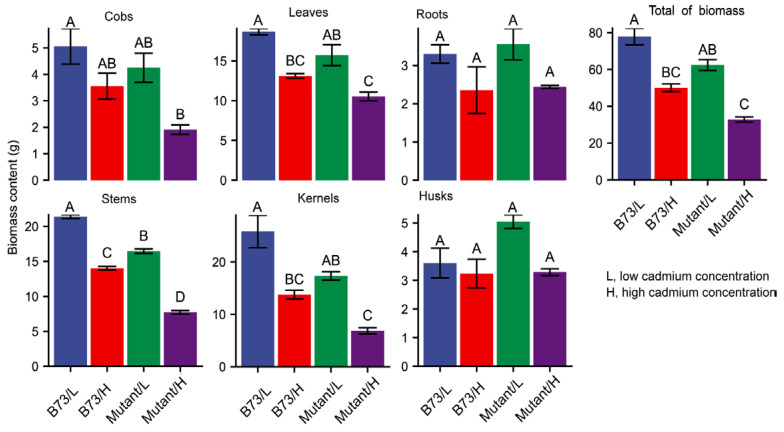
The biomass content in different organs of B73 and the mutant under diverse Cd stress conditions. Mutant represents the genotype *ZmHMA3*, and Cd represents cadmium. The upper letter in each column indicates the difference derived from Duncan’s multiple range test.

Excessive levels of the heavy metal Cd can inflict substantial damage on plant growth and development. No significant differences in root biomass were observed between the two soil Cd treatments in B73 and *ZmHMA3* (Figure 2). However, under high-Cd soil conditions, the aboveground growth of both B73 and *ZmHMA3* was significantly inhibited, with *ZmHMA3* experiencing a greater reduction in biomass compared to B73. This implies that the elevated Cd content in plants has a pronounced inhibitory effect on biomass accumulation. Relative to the low-Cd treatment, the dry weights of the stems, leaves, and grains decreased by 34.4%, 29.9%, and 46.7% in B73 and 53.0%, 33.0% and 60.4% in *ZmHMA3*, respectively (Figure 2). Notably, the Cd content in the grains was the lowest, but its biomass was most affected by Cd stress. This suggests that maize grain yield is more sensitive to Cd stress, emphasizing its susceptibility to adverse impacts under elevated Cd concentrations.

### 3.3. Effect of Soil Cd Concentration on Physiological Performance

**Figure 3:**
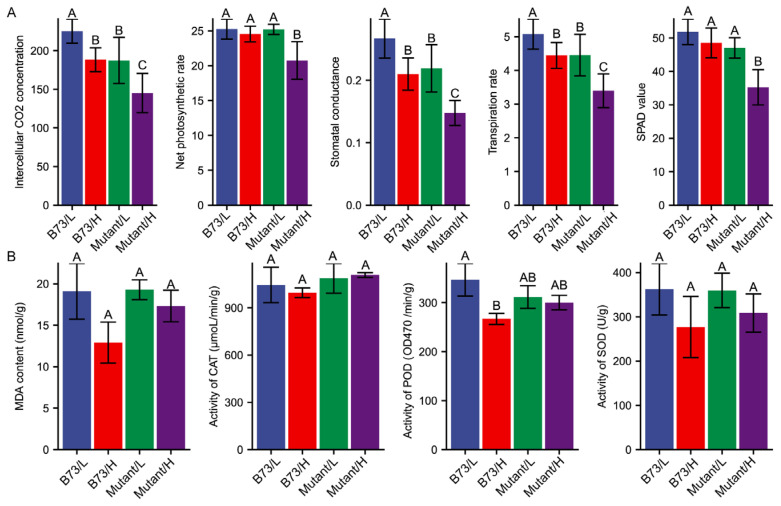
The physiological response to Cd stress of B73 and the mutant. (**A**) The response of photosynthetic parameters under different Cd stress conditions. (**B**) The response of antioxidant enzymes under different Cd stress conditions. L represents the low-Cd condition, H represents the high-Cd condition, Mutant represents the genotype *ZmHMA3*, and Cd represents cadmium. The upper letter in each column indicates the difference derived from Duncan’s multiple range test.

Leaves exhibited the highest cadmium content among the organs studied. Assessing photosynthetic parameters is crucial for understanding the physiological responses of leaves to Cd stress. To investigate the impact of mutation-induced photosynthesis under different Cd stress conditions, we measured the photosynthetic parameters for the ear leaves in B73 and *ZmHMA3* (Figure 3A). The net photosynthetic rate (Pn), reflecting the plant’s ability to assimilate CO_2_ and its foundation for growth and development, slightly decreased in B73 with the increasing soil Cd content, though the difference was not significant. In *ZmHMA3*, the net photosynthetic rate decreased by 17.7%, and the difference was highly significant. Stomatal conductance, intercellular CO_2_ concentration, and transpiration rate were significantly lower in both B73 and *ZmHMA3* under the high-Cd treatment compared to the low-Cd treatment. Plant chlorophyll content, indirectly reflected by the SPAD value, showed a positive correlation with the chlorophyll content. As soil Cd content increased, chlorophyll synthesis was significantly inhibited. The difference in chlorophyll content between the high- and low-Cd treatments in B73 was not significant, whereas in *ZmHMA3*, it was statistically significant (*p* < 0.05). These findings indicate that Cd stress reduces the chlorophyll content, decreases the stomatal aperture, lowers the intercellular CO_2_ concentration, and inhibits the transpiration rate, leading to a decrease in net photosynthetic rate, ultimately affecting plant growth and development. In comparison to the high-Cd-accumulating *ZmHMA3*, B73 is less affected by Cd stress and can maintain a higher photosynthetic rate, ensuring normal plant growth.

### 3.4. Antioxidant Enzymes Responses to Cd Stress

Malondialdehyde (MDA) content, a product of lipid peroxidation, serves as a biomarker for oxidative damage and cellular injury, reflecting the toxicity of Cd to plants. In high-Cd-stressed grains of B73, the amount of MDA decreased significantly (*p* < 0.05) compared with the low-Cd treatment. Conversely, the MDA content in *ZmHMA3* decreased slightly with no statistical difference between the high- and low-Cd treatments (Figure 3B). This decrease in MDA content might be attributed to an increase in antioxidant enzyme activity. Subsequently, we measured the enzymatic activities of CAT, SOD, and POD in maize grains (Figure 3B). The differences in CAT and SOD activity between the two treatments was not statistically significant in both genotypes. CAT activity slightly decreased in high-Cd-stressed grains of B73 (decreased by 4.68%), while CAT activity in *ZmHMA3* grains increased slightly compared with the low-Cd treatment (increased by 1.89%). SOD activity decreased slightly in high-Cd-stressed grains of both genotypes, but Cd-induced SOD activity was more noticeable in B73 (23.61%) than in *ZmHMA3* (14.21%). Significant changes in POD activity were observed between the high- and low-Cd treatments. POD activity in B73 was significantly lower (decreased by 23.05%) under high-Cd treatment than under low-Cd treatment (*p* < 0.01), but only slightly decreased in *ZmHMA3* (decreased by 3.64%). These results indicate that oxidative damage caused by Cd toxicity was more pronounced in *ZmHMA3* than in B73. The greater protection in grains of the high-Cd-accumulating genotype *ZmHMA3* is achieved through a more efficient reactive oxygen species (ROS)-scavenging machinery, as evidenced by the maintenance of POD activity under high Cd stress. POD activity plays an essential role in preventing oxidative damage caused by Cd toxicity in maize grains.

### 3.5. Proteomic Analysis of Kernels in Response to Cd Stress

**Figure 4:**
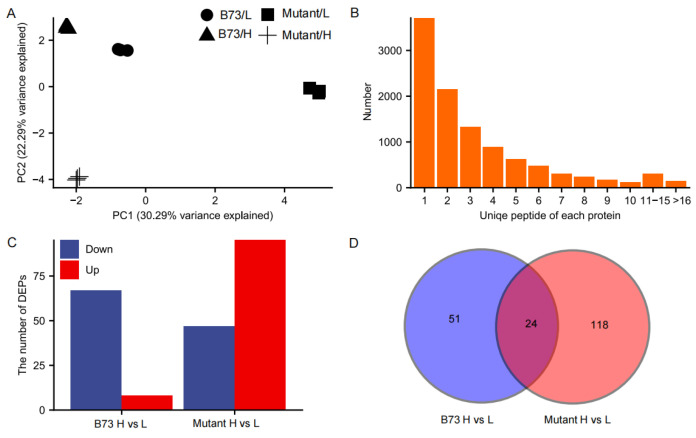
Proteomic characterizations of maize kernels under Cd-stressed conditions. (**A**) Statistical analysis of the number of proteins with unique peptides. (**B**) Principal component analysis of all quantified samples. (**C**) The number of DEPs in B73 and the mutant under Cd stress conditions. (**D**) Venn diagram showing the number of common and unique DEPs in B73 and the mutant. L represents the low-Cd condition, H represents the high-Cd condition, Mutant represents the genotype *ZmHMA3*, Cd represents cadmium, and DEPs represents differentially enriched proteins.

Quantitative protein analysis was employed to unravel the molecular mechanism underlying the difference in Cd accumulation in grains of B73 and *ZmHMA3* under varying Cd stress conditions. A total of 10,487 proteins were identified with a 1% false discovery rate (FDR) among 119,196 distinct peptides derived from 263,872 spectra. Of these proteins, approximately 64.62% had more than two unique peptides (Figure 4A). Pearson correlations among the four treatments in the three replicates ranged from 0.41 to 0.61 (Figure S1). The principal component (PC) analysis results demonstrated a clear separation, with PC1 and PC2 explaining 30.29% and 22.29% of the protein expression variations among 12 kernel samples (Figure 4B), indicating the significant impact of Cd treatment on the proteome in both genotypes.

Protein expression profiles in both genotypes were significantly altered by the high-Cd treatment compared with the low-Cd treatment, leading to differential regulation. Applying a threshold value of fold change >1.3 (up-regulated) or <0.77 (down-regulated) at *p* < 0.05, a total of 193 DEPs were identified between the two genotypes under different Cd soil conditions. Among these DEPs, 8 and 67 proteins were upregulated and downregulated, respectively, in B73, while 95 and 47 proteins were upregulated and downregulated in *ZmHMA3*, respectively, with 24 common DEPs (Figure 4C,D). Among the common DEPs, one and six were upregulated and downregulated in both genotypes, respectively. Notably, 17 proteins were identified as being upregulated in *ZmHMA3* but downregulated in B73 (Table S1), suggesting their pivotal roles in Cd accumulation. Furthermore, 51 DEPs were unique to B73 and 118 DEPs were unique to *ZmHMA3*, indicating significant changes in protein expression in the two genotypes potentially involving a range of physiological and biochemical processes.

### 3.6. GO Enrichment Analyses of DEPs

**Figure 5:**
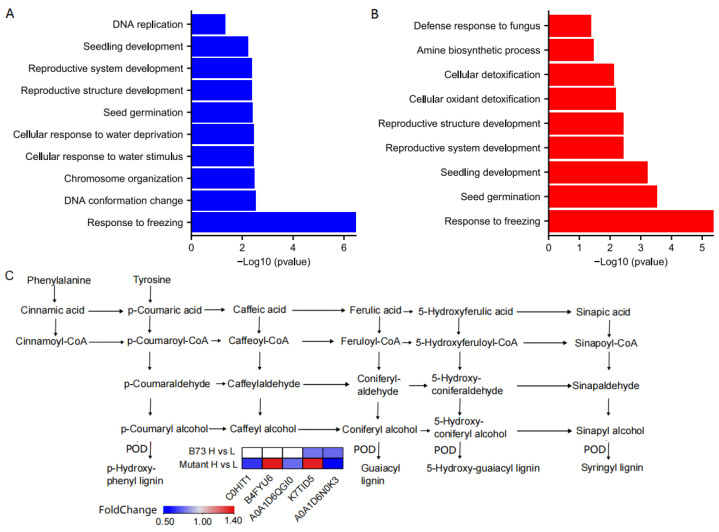
The functional enrichment analysis of DEPs in B73 and the mutant. (**A**) The significant GO terms of biological processes involved in the DEPs in B73. (**B**) The significant GO terms of biological processes involved in the DEPs in the mutant. (**C**) The metabolic pathway of lignin biosynthesis. The heatmap shows the 5 DEPs catalyzed in the final step. L represents the low-Cd condition, H represents the high-Cd condition, Mutant represents the genotype *ZmHMA3*, and DEPs represents the differentially enriched proteins.

We conducted GO annotation analysis on the DEPs to unravel their potential biological functions. The DEPs were categorized into three main GO classes: biological processes (BP), molecular functions (MF), and cellular components (CC). B73 and *ZmHMA3* exhibited significant enrichment with 28 and 34 DEPs in the GO classification, respectively. Twenty-five and twenty-four significantly enriched GO units were observed in B73 and *ZmHMA3*, respectively (Figure 5A,B, Figures S2 and S3). In B73, the 10 significantly enriched GO units in BP included response to freezing, DNA conformation change, chromosome organization, cellular response to water stimuli, cellular response to water deprivation, seed germination, reproductive structure development, reproductive system development, seedling development, and DNA replication (Figure 5A). The eight significantly enriched GO units in MF comprised IgE binding, immunoglobulin binding, DNA helicase activity, catalytic activity, acting on DNA, DNA binding, heterocyclic compound binding, organic cyclic compound binding, and protein-containing complex binding (Figure S2). The seven significantly enriched GO units in CC encompassed monolayer-surrounded lipid storage bodies, lipid droplets, chromosomes, intracellular non-membrane-bounded organelles, non-membrane-bounded organelles, chromatin, and nuclear chromatin (Figure S3). In *ZmHMA3*, the nine significantly enriched GO units in BP included response to freezing, seed germination, seedling development, reproductive system development, reproductive structure development, cellular oxidant detoxification, cellular detoxification, amine biosynthetic process, and defense response to fungi (Figure 5B). The eight significantly enriched GO units in MF comprised immunoglobulin binding, IgE binding, peroxidase activity, UDP-glucosyltransferase activity, quercetin 7-O-glucosyltransferase activity, quercetin 3-O-glucosyltransferase activity, double-stranded RNA binding, and antioxidant activity (Figure S4). The seven significantly enriched GO units in CC included lipid droplets, aleurone grains, monolayer-surrounded lipid storage bodies, aleurone grain membranes, protein storage vacuoles, storage vacuoles, and endoplasmic reticulum lumens (Figure S5). In terms of BP, both genotypes showed enrichment in seed germination, seedling development, reproductive system development, and reproductive structure development, with the latter two being the most prominent. For both genotypes, most DEPs were involved in reproductive system development and reproductive structure development, indicating their fundamental roles in Cd stress resistance in maize grains. Regarding MF, both genotypes exhibited significant enrichment in IgE binding and immunoglobulin binding. The DEPs in B73 were mainly related to heterocyclic compound binding and organic cyclic compound binding, while those in *ZmHMA3* were mainly associated with antioxidant enzyme activity and glucosyltransferase activity. This suggests that higher Cd accumulation might affect enzyme activity in maize grains, indicating distinct response patterns to Cd stress between the two genotypes. Among the CC process functions, monolayer-surrounded lipid storage bodies and lipid droplets were commonly enriched in both genotypes. DEPs in B73 were predominantly associated with intracellular non-membrane-bounded organelles and non-membrane-bounded organelles, highlighting the importance of non-membrane-bounded organelles in B73’s tolerance to Cd stress.

### 3.7. KEGG Signal Pathway Enrichment for DEPs

By scrutinizing the significantly enriched metabolic pathways of DEPs, valuable insights can be obtained into the metabolic regulatory pathways undergoing substantial changes under diverse Cd stress conditions. This analysis facilitates the identification of the metabolic processes most affected by Cd stress, offering a deeper understanding of the molecular mechanisms guiding the response of maize grains to Cd stress. According to the KEGG enrichment analysis, three and nine DEPs were significantly enriched in B73 and *ZmHMA3*, respectively (Tables S2 and S3). The KEGG enrichment analysis revealed that DEPs in B73 were particularly enriched in amino sugar and nucleotide sugar metabolism, whereas those in *ZmHMA3* were enriched in phenylpropanoid biosynthesis, nitrogen metabolism, and glyoxylate and dicarboxylate metabolism. Specifically, in amino sugar and nucleotide sugar metabolism, three DEPs were down-regulated in B73, including acidic endochitinase (A0A1D6HTN9), chitinase (B4FTS6), and chitinase chem5 (B4G1T3). Notably, pentatricopeptide repeat protein-like (B4FYX5) and glutamine synthetase (B4FMX4), associated with nitrogen metabolism and glyoxylate and dicarboxylate metabolism, were significantly enriched in *ZmHMA3*. In the KEGG pathway enrichment analysis, phenylpropanoid biosynthesis stood out as being significantly enriched in both the up-regulated and down-regulated DEPs of *ZmHMA3* (Figure 5C). The up-regulated DEPs included peroxiredoxin (B4FYU6), peroxidase (K7TID5), and β-glucosidase (A0A1D6JAB3). The down-regulated DEPs were peroxidase (C0HIT1, A0A1D6QGI0 and A0A1D6N0K3).

### 3.8. Protein–Protein Interactions Analysis

**Figure 6:**
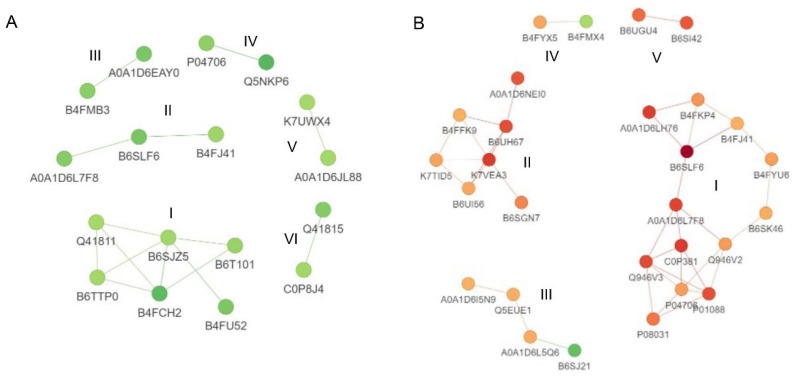
Protein–protein interaction analysis of DEPs in maize grains. (**A**) Protein–protein interaction analysis of DEPs in B73. (**B**) Protein–protein interaction analysis of DEPs in *ZmHMA3*. The circles represent the DEPs, and different colors indicate the up/down-regulation of DEPs (green for downregulated and red for upregulated). The intensity of the color corresponds to the magnitude of the fold change. DEPs: differentially expressed proteins.

To obtain a comprehensive understanding of the response to Cd stress in B73 and *ZmHMA3*, a protein–protein interaction map of DEPs was generated, considering confidence scores higher than 0.7 (Figure 6). In the protein–protein interaction of B73, 17 down-regulated DEPs formed 2 networks and 4 pairs of interacting proteins (Figure 6A). The largest group (cluster I) consisted of six histone proteins. In *ZmHMA3*, 3 networks and 2 pairs of interacting proteins were observed, comprising 26 up-regulated and 2 down-regulated DEPs (Figure 6B). The top two groups (cluster I and Cluster II) included 13 and 7 up-regulated DEPs, predominantly associated with biotic or abiotic stress and nutrient storage. Additionally, two protein pairs were identified to be involved in stress response (Cluster IV) and nutrient storage (Cluster V).

## 4. Discussion

Cadmium (Cd) poses a threat as a toxic heavy metal and a major environmental contaminant in central-southern and southwest China, potentially compromising crop yield and quality [37]. Mitigating Cd accumulation in maize grain is essential to reduce Cd toxicity risks in the food chain, safeguarding human health. In our previous study, a non-functional variant of *ZmHMA3*, a member of the heavy metal ATPases (HMAs), was identified as a key player in regulating high Cd accumulation in maize grain [17]. Additionally, *ZmHMA2* and *ZmHMA3* were significantly associated with leaf Cd concentration under various Cd levels, as revealed by genome-wide association analysis [38]. Despite this, the mechanisms underlying Cd accumulation and stress response in *ZmHMA3* remain incompletely understood. Therefore, a deeper exploration is necessary to elucidate the distinctions in Cd accumulation differences between the genetic variations of *ZmHMA3*.

The translocation of toxic metals from the roots to the shoots is a key process governing their accumulation in the aboveground tissues [39,40]. Previous studies have suggested that HMA3 genes influence Cd sequestration in the roots, preventing Cd translocation and accumulation from the roots to the aboveground parts [13]. Our investigation into Cd content across different organs in Cd-polluted soils revealed that *ZmHMA3* accumulated more Cd than that of B73, particularly in the aboveground tissues (Figure 1), indicating higher Cd translocation from the roots to the aboveground tissues in *ZmHMA3*.

Excessive Cd in plants leads to morphological and physiological changes, ultimately resulting in decreased plant biomass [41]. The impact on plant growth is more pronounced with increasing Cd content, especially in *ZmHMA3* with a high Cd accumulation ability. The greater suppression in *ZmHMA3* compared to B73 under Cd stress could be attributed to the significantly decreased plant biomass (Figure 2). A higher level of Cd concentration was observed in the leaves than in other organs. The leaves were severely affected by elevated Cd concentrations, and excess Cd might affect photosynthesis and transpiration [42]. In our study, the net photosynthetic rate, stomatal conductance, intercellular CO_2_ concentration, and transpiration rate were significantly inhibited in *ZmHMA3* (Figure 3). Previous research has demonstrated that Cd toxicity has the ability to inhibit chloroplast synthesis and directly influence photosynthetic efficiency [43]. The observed decrease in SPAD values in *ZmHMA3* under high-Cd treatment further emphasizes the adverse effects on photosynthetic efficiency.

Typically, kernel Cd content is lower compared to that of other organs [33]. The variations in grain Cd concentration between genotypes were significantly greater than in other organs. Employing TMT-based quantitative proteomics analysis, we identified a total of 10,487 proteins, with only 75 and 142 DEPs identified in B73 and *ZmHMA3*, respectively (Figure 4). The predominant down-regulation of DEPs in B73 and up-regulation in *ZmHMA3* underline distinct patterns of protein expression patterns in response to varying levels of Cd stress. Key proteins, including A0A1D6F7P8 (ribosome-associated membrane protein RAMP4), B4FFU8 (GDSL esterase/lipase), P81009 (Defensin-like protein 2), and B6SXF2 (defensin-like protein 6), were found to be up-regulated under Cd stress. Ribosome-associated membrane protein (RAMP4) has been documented to stabilize the integrity of membrane proteins under Cd stress [44,45]. Most GDSL esterase/lipase family genes play a role in enhancing adaptability to Cd stress [46]. Defensin-like proteins also respond to Cd stress, and it has been reported that *CAL1* encodes a defensin-like protein that positively regulates Cd accumulation in rice leaves. *CAL1* acts by chelating Cd in the cytosol and promoting Cd secretion into the extracellular spaces, thereby reducing cytosolic Cd concentration while facilitating long-distance Cd transport via xylem vessels [16]. Our results imply that these four proteins potentially participate in maize kernel responses to Cd stress and play a role in reducing Cd content, and enhance adaptability under Cd stress.

Through GO annotation and KEGG enrichment of DEPs, we observed significant impacts of Cd stress on amino sugar and nucleotide sugar metabolism, phenylpropane biosynthesis, nitrogen metabolism, and glyoxylate and dicarboxylate metabolism. After the entry of Cd into cells, lignin, an important component of the cell wall, can bind with Cd to sequester it in the cell wall and decrease the amount of Cd entering the cell interior, thereby mitigating the toxic effects of Cd [47]. Previous studies have shown that the activation of phenylpropanoid compound biosynthesis (e.g., lignin) may represent a crucial pathway for successful detoxification under Cd stress [48]. Phenylpropanoid biosynthesis was significantly enriched in both the up-regulated and down-regulated DEPs, with five DEPs being associated with lignin biosynthesis, which produces lignin as an end-product in vascular plants (Figure 5). Additionally, under Cd stress, the increased enzyme activity of POD triggers lignin production, thereby enhancing Cd tolerance [49]. The expression of POD genes plays a crucial role in mitigating Cd-induced oxidative stress, helping to maintain reactive oxygen species (ROS) balance in grains and ensuring normal cell function [50]. In the present study, genes encoding peroxiredoxin, peroxidase, and β-glucosidase have a potential role in decreasing Cd-induced ROS overaccumulation, which may be the reason for the differences in Cd accumulation in maize grains.

## 5. Conclusions

This study presents a comprehensive analysis of phenotypic and proteomic differences between wild-type B73 and its high-Cd-accumulation mutant *ZmHMA3* under varying Cd soil conditions. *Zmhma3* exhibited higher Cd accumulation in different organs, particularly in the aboveground tissues, indicating strong Cd transport ability from the roots to aboveground. Cd-induced toxicity led to oxidative damage, inhibited photosynthetic efficiency, and reduced biomass accumulation, with *ZmHMA3* experiencing a more pronounced effect compared to B73. Proteomic analysis identified numerous maize kernel DEPs responding to Cd stress, with a greater number of upregulated genes in *ZmHMA3*. This study sheds light on the differences in Cd accumulation in maize grains, providing insights into potential candidate genes for low Cd accumulation.

## Data Availability

The datasets and materials presented in the investigation are not publicly available due to privacy concerns but will be available from the corresponding author.

## Supplementary Materials

The following supporting information can be downloaded at: https://www.mdpi.com/article/10.3390/genes14122204/s1, Figure S1: Pearson correlations of all samples under diverse Cd stress conditions; Figure S2: The significant GO terms of molecular functions involved by DEPs in B73; Figure S3: The significant GO terms of cellular components involved by DEPs in B73; Figure S4: The significant GO terms of molecular functions involved by DEPs in mutant; Figure S5: The significant GO terms of cellular components involved by DEPs in mutant; Table S1: The information of 24 common differentially enriched proteins in B73 and the mutant; Table S2: The KEGG pathways associated with differentially enriched proteins in B73; Table S3: The KEGG pathways associated with differentially enriched proteins in the mutant.
