# Supplementary material for: Phenotypic and Proteomic Insights into Differential Cadmium Accumulation in Maize Kernels
Source: Genes (Basel). 2023 Dec 13;14(12):2204. doi: 10.3390/genes14122204 (PMC10742529; doi:10.3390/genes14122204)
Supplement: Supplementary file 1 [file genes-14-02204-s001.zip › Figure S1-5.pdf]

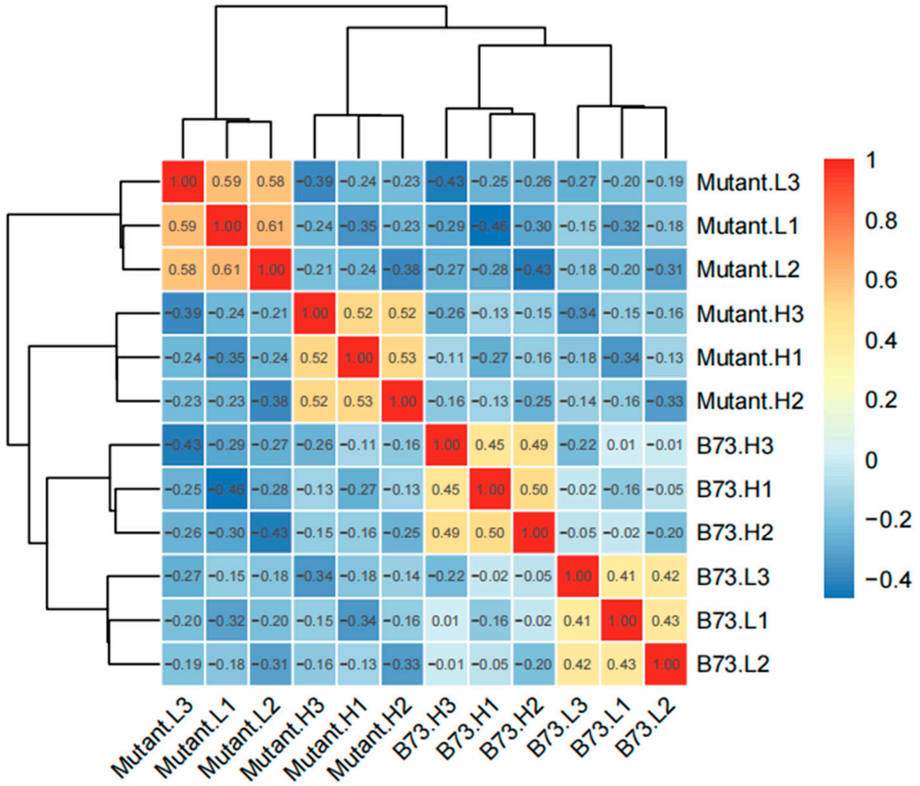

Figure S1. Pearson correlations of all samples under diverse Cd-stress conditions. L represents low-Cd condition, H represents high-Cd condition, Mutant represents genotype *zmhma3*, Cd represents cadmium, and the number indicates the replicates.

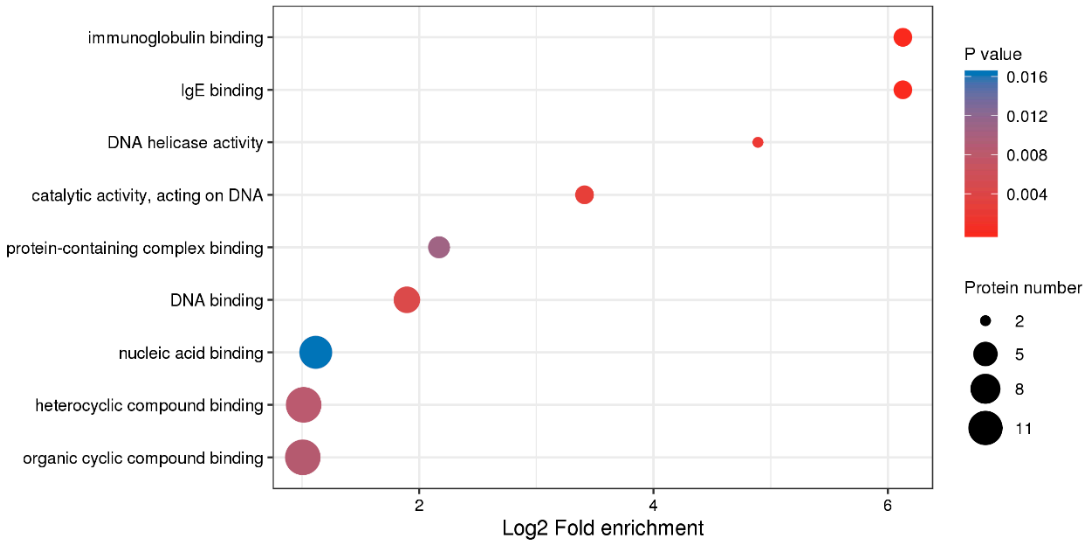

Figure S2. The significant GO terms of molecular function involved by DEPs in B73.

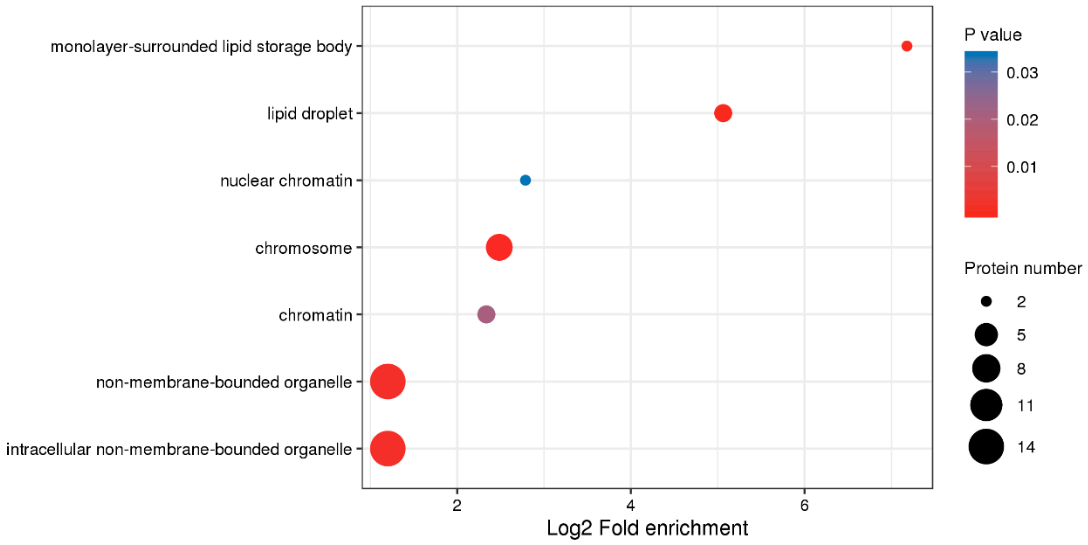

Figure S3. The significant GO terms of cellular component involved by DEPs in B73.

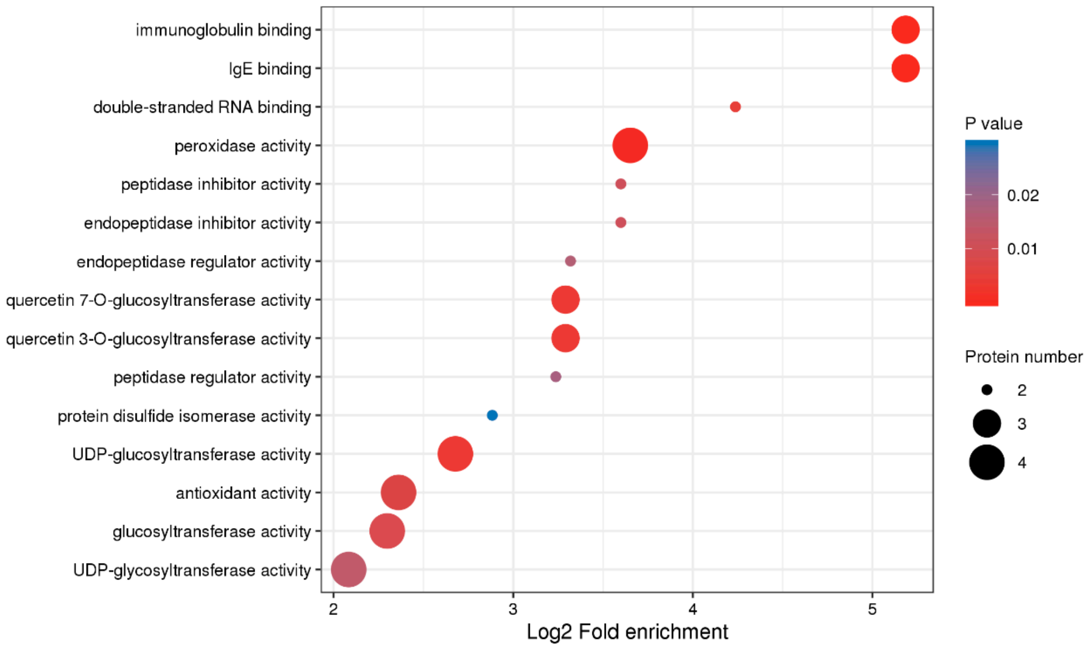

Figure S4. The significant GO terms of molecular function involved by DEPs in mutant.

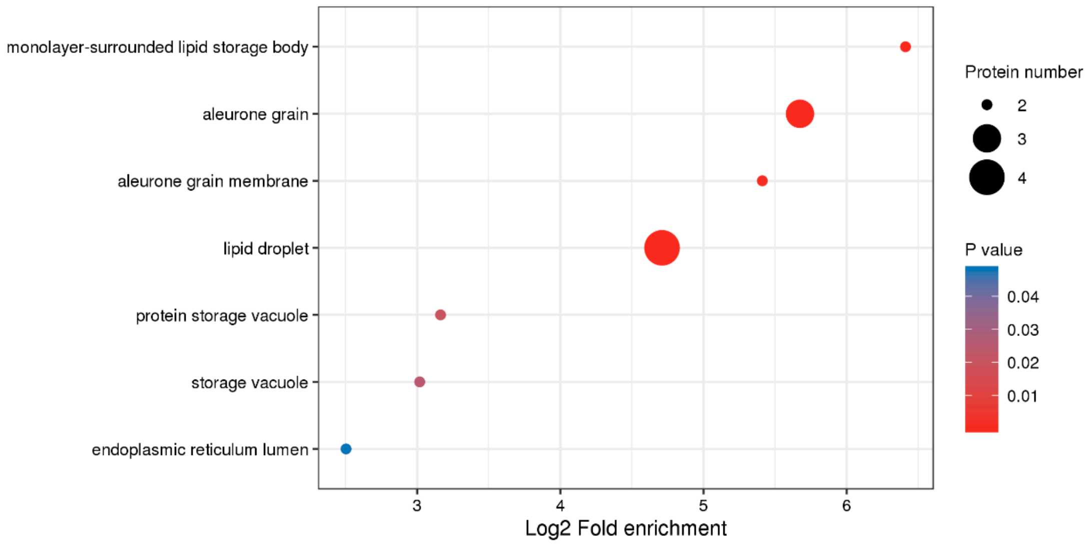

Figure S5. The significant GO terms of cellular component involved by DEPs in mutant.
